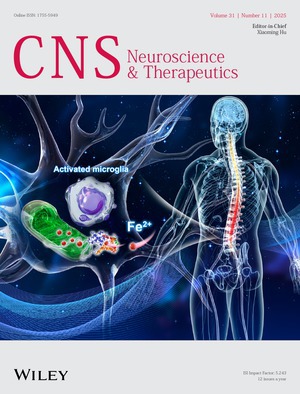# Front Cover

**DOI:** 10.1111/cns.70664

**Published:** 2025-11-21

## Abstract

The cover image is based on the article *Microglial SLC25A28 Knockout Mitigates Spinal Cord Injury in Mice by Inhibiting Heme Synthesis and Subsequent NOX2 Activation* by Huangtao Chen et al., https://doi.org/10.1111/cns.70638.